# Evaluation of Workability and Structuration Rate of Locally Developed 3D Printing Concrete Using Conventional Methods

**DOI:** 10.3390/ma15031243

**Published:** 2022-02-08

**Authors:** Sara Ahmed, Sherif Yehia

**Affiliations:** Civil Engineering Department, American University of Sharjah, Sharjah 26666, United Arab Emirates; syehia@aus.edu

**Keywords:** 3D printing, workability, structuration rate, buildability, aggregate-to-binder ratio, compressive strength

## Abstract

Concrete 3D printing is a novel construction method that can bring new horizons to the construction industry. However, there are still many challenges that limit its capabilities. Despite the huge research efforts, to date, there are still no standardized acceptance criteria and guidelines for the evaluation of printing concrete. Therefore, the main objective of this research was to develop 3D printing mixes with different aggregate-to-binder (a/b) ratios (1.2, 1.5, and 1.8) and evaluate it in terms of its fresh printing properties, which include the workability, extrudability, setting time, open time, and buildability. The compressive strengths of cast and printed specimens were also tested to determine the effect of the layering process. The workability was evaluated using commonly used devices in the construction industry (slump and flow table test) and was monitored over time along with the penetration test to indicate the structuration rate of concrete. From the experimental results and observations, the flow test resulted in the best indication of the structuration rate (thixotropy) of concrete, followed by the penetration and slump tests. The a/b ratio affected all the investigated properties of the printing concrete. Higher a/b ratios resulted in increased structuration rate, buildability, and compressive strength of cast specimens. However, for printed specimens, the compressive strength decreased with the increase in a/b ratio due to increased thixotropy. Therefore, from the results of the present investigation, it can be concluded that high a/b ratios (>1.5) are not desirable for printing concrete.

## 1. Introduction

The versatility, durability, and economy of concrete make it the most widely used building material ever since ancient Egypt. In addition to the three main components of concrete—aggregates, cement, and water—other materials such as additives could always be utilized to proportion the concrete to meet specific requirements. However, the use of conventional concrete involves formwork usage and makes the construction industry quite challenging. For example, the construction industry is a high-risk industry that involves the death and injury of many workers. In 2019, the Census of Fatal Occupational Injuries (CFOI) reported that the number of fatal work injuries reached up to 5333 in the United States with a worker dying every 99 min due to work-related injuries [1]. Cement, which compromises about 10–15% in concrete, is considered the third-largest source of anthropogenic carbon dioxide (CO_2_) after fossil fuel and land use, contributing to around 5% of the global anthropogenic CO_2_ emissions [2,3]. Additionally, the use of formworks in construction is demanding due to the limited availability of different sizes and shapes, the time required for formwork setup, and the number of times the formwork material can be reused.

Over the past decades, new innovative technology has been gaining ground, known as additive manufacturing. This technology has the potential to solve many of the problems the construction industry is facing such as those aforementioned. When compared to the conventional process, 3D printing technology may reduce the number of laborers in the construction field, may reduce construction time and materials used, and may eliminate formwork usage [3,4,5,6]. This consequently will reduce the overall costs of the construction project and will lead to the expansion of a sustainable environment with a larger variety of customized homes and buildings. Some of the structural applications of the 3D printing technology include: a five-story building completed in 45 days in China in 2015 [7], the world’s first 3D printed office in Dubai completed in 2016 [7,8,9], and the world’s largest two-story building in Dubai completed in 2019 [10]. The erection of the two-story building was considered a turning point in the construction industry where the wastes generated and construction costs were reported to be 60% less and the workers involved were 50% lower than the conventional methods [10]. Such advantages make printing concrete a promising opponent for ordinary concrete and/or self-consolidating concrete (SCC) in architectural engineering applications.

Although this technology has remarkable benefits, there are still many challenges that restrict its application. In general, the material used for 3D printing should be designed to meet the requirements of the layering process and should be extrudable and buildable with strong interlayer strength. The challenges associated with the printing concrete were recently highlighted in [11] and include low bond strength between the layers, deformation and appearance complexities that result from a free-form system, drying shrinkage, and plastic deformations. Despite these challenges, the development of 3D printing concrete is, fortunately, less challenging to date due to the present state of knowledge of a vast range of materials that facilitate the design of such material. However, the real concern is the absence of universal test standards and acceptance criteria to evaluate the free-form construction concrete. Moreover, there is a lack of knowledge and guidelines in the mixing procedure required for the production of the 3D printing concrete.

Le et al. [5] conducted one of the earliest studies in 2011 that provided an initial understanding of the fresh properties of the printing concrete. The extrudability and buildability were the two main criteria that should be satisfied by adjusting the workability. In [5], it was pointed that workability conventional test methods such as the slump do not provide fundamental physical properties, and tests that provide a more rheological investigation should be selected. Thus, the workability was tested using the shear vane apparatus. Since then, several studies [12,13,14] have attempted to evaluate the workability using either conventional methods or other less commonly used methods to study the rheology of the printing concrete. 

Rheology in general is the science of studying the flow of a matter and its deformation under shear stress [15]. Its basic parameters include yield stress, viscosity, and thixotropy. The minimum stress required to initiate flow is the yield stress and is considered as the key parameter to determine the stability of the layer. Viscosity is the resistance of a material to change in shape or flow, while thixotropy results from the time the cementitious materials require to rearrange the microstructure of the paste after the introduction of shear energy [15,16]. Unlike self-consolidating concrete, which requires low yield stress, the 3D printing material needs to be of high yield stress, low viscosity, and high thixotropy. 

Malaeb et al. [12] evaluated the fresh printing concrete properties and tested the flowability using the slump-flow test, and the range that satisfied both the extrudability and buildability was found to be within 1.0–2.0 cm/s. Ma et al. [14] tested the flowability using the slump test, V-funnel test, and jumping table test of concrete developed using partial replacement of copper tailings. The slump and spreading diameter that maintained printability were found to be 88–32 mm and 175–210 mm, respectively. The V-funnel test showed a good indication of the viscosity of the concrete. Perrot et al. [17] developed a model that predicts the time at which the layers collapse by determining the yield stress and vertical stress of fresh concrete. 

Zhang et al. [18] investigated the yield stress, viscosity, and thixotropy of concrete developed with different sand to binder ratios (0.6, 0.8, 1.0, 1.2, and 1.5) using a concrete rheometer. The acceptable ranges for pumpability and extrudability were found to be 178.5–359.8 Pa for the yield stress, 3.8–4.5 Pa/s for the viscosity, and 6284.5 Pa/s for the thixotropy. Moreover, an initial diameter of 192.5–269 mm was found to be optimal for the layer to maintain its shape. Panda et al. [19] investigated the effect of using one-part geopolymers on the fresh rheological concrete properties. Geopolymers are used as binders and are a more sustainable alternative than cement. It was found that geopolymers provide a material with high yield stress and low viscosity, which is very beneficial for printing concrete. The durability and challenges of geopolymer concrete containing waste glass were reviewed by Siddika et al. [20]; however, the review shows that no major investigations have been conducted on the durability of such concrete and therefore additional research is required in this area. In general, the durability of 3D printing concrete is still in its infancy and remains ambiguous, and therefore tests such as those noted in [21] are necessary for its assessment. Sooryanarayana et al. [22] evaluated the yield stress of coarse aggregate printing concrete using an international center for aggregate research concrete rheometer. The results of the study showed that the use of coarse aggregate increases the yield stress of paste due to greater physical interlock between the aggregates as compared to the fine aggregates. Table 1 summarizes some of the optimal ranges that satisfy the extrudability (printability) using different workability test methods. 

On the basis of the available information from the literature, it is conspicuous that there is still no standard method and/or acceptance criteria to evaluate the workability of the printing concrete. Other tests to measure the yield stress as noted in [25] include stress ramp, creep test series, and oscillation stress sweep. However, such tests require preceding knowledge to set the range of measurement parameters. Therefore, further research is crucial to standardize the evaluation methods and acceptance criteria. Although evaluations in [19,21] attempted to use concrete rheometers to evaluate the workability, it is indicated that some of the commercially available rheometers may not provide enough torque for viscosity measurement of high-viscous materials. Different rheometers also tend to produce different results. In addition, the vane may rotate in the center of the concrete to create the so-called ‘plug’ [26]. These uncertainties make some of the currently available results controversial. This emphasizes the need for the use of more systemized approaches for workability evaluation.

In general, conventional test methods such as the slump and flow table test are preferable on-site for their ease of use. Flow test, in particular, may provide a good indication of the rheology of the concrete as it measures the resistance of concrete flow within a specific time interval. In fact, it was previously shown that a relationship exists between the rheological parameters and the flow table test [15,24]. Most of the studies [5,12,13,19] only report the range of workability at which extrudability and buildability are satisfied; however, to the authors’ knowledge, hardly any studies provide the rate at which workability is lost over time [27]. The rate at which concrete loses its workability will evidently give an indication of the rheology and structuration rate of concrete [28]. 

Therefore, from the conducted review, the main objectives of the current investigation are as follows:

- Develop 3D printing concrete utilizing available construction materials and evaluate its workability using commonly used devices in the construction industry (slump and flow tests) to determine the ranges at which extrudability and buildability are satisfied.
- Study the structuration rate of concrete using simple devices such as the slump, flow, and penetration tests. The results of the present investigation are expected to encourage researchers to use and explore simple equipment that can facilitate and standardize the evaluation of fresh printing concrete in the future.
- Study the effect of structuration rate on certain properties of printing concrete such as buildability and compressive strength.

To achieve the main objectives of the study, printing concrete was produced using conventional mixing equipment at normal speeds. The a/b ratio is one of the factors that affects the structuration rate (thixotropy) of concrete and thus three main mixes were developed in this study with different a/b ratios of 1.2, 1.5, and 1.8 to assess its effect. The evaluated properties included the workability, extrudability, setting-time, open time, buildability, and compressive strength of both cast and printing specimens. Pearson correlation analysis was performed to determine which of the above-mentioned devices can best predict the structuration rate.

## 2. Experimental Program

The experimental investigation involved two phases of evaluation. Phase 1 involved mix optimization while phase 2 involved property evaluation of printing concrete. In phase 1 evaluation, two groups of mixes were developed to evaluate the effect of different supplementary cementitious materials (SCM) on the extrudability of the printing concrete as well as the compressive strength of cast specimens. The optimal mix of the two groups was selected and developed with three different aggregate-to-binder (a/b) ratios, and the setting-time of the three mixes were determined. Phase 1 is considered only a preliminary stage and, thus, the mixes were produced on a small scale using a Hobart mixer. For phase 2 evaluation, the main objective was to evaluate the effect of different aggregate to binder (a/b) ratios on the fresh printing properties. The evaluation criteria included the extrudability, setting-time, open-time, workability, and buildability, as well as the compressive strength of cast and printed specimens to indicate the concrete strength. Since this stage involved the evaluation of several properties, a conventional concrete mixer was used for the production of a larger-scale mixing to satisfy volume requirements. 

**Figure 1:**
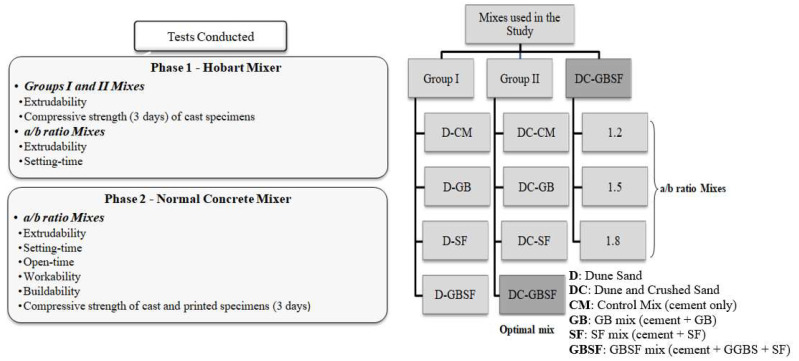
Summary of conducted tests and mixes used in the study.

Figure 1 summarizes the experimental program conducted in the current study. The two groups (Group I and Group II), in phase 1, consisted of four mixes. Group I mixes were prepared with dune sand only, while Group II mixes were prepared with a blend of dune and crushed sand. The four mixes of the two groups consisted of a mix prepared with ordinary Portland cement only (control mix CM); cement and ground granulated blast furnace slag (GB); cement and silica fume (SF); and cement, GB, and SF combined (GBSF). The GB and SF were used as a partial replacement of cement by weight. The mixes were labeled to identify the two groups of mixes of the fine aggregate and cement/cementitious materials used. The references were given such that the first one or two letters refer to the type of sand used either dune sand (D), or dune and crushed sand combined (DC); these letters were then followed by a hyphen to indicate the type of binder used in the mix. CM for control mix consisting of Portland cement only. For instance, DC-GBSF refers to the Group II mix, which has fine aggregates consisting of dune and crushed sand and binding material of cement, GB, and SF combined. The optimum mix of the two groups was selected and developed with a/b ratios of 1.2, 1.5, and 1.8 for the rest of the evaluation. These mixes were referred to as 1.2, 1.5, and 1.8 mixes, reflecting the different a/b ratios. 

### 2.1. Materials

**Figure 2:**
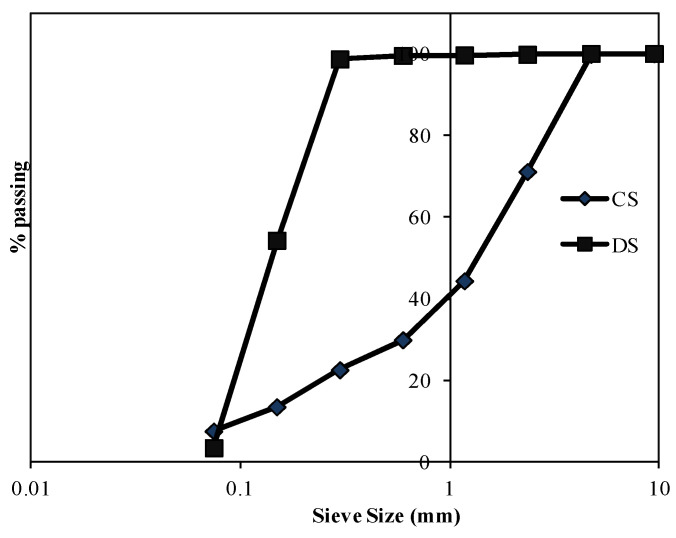
Dune and crushed sand particle size distribution.

CEM-I 42.5-R0 and GB complying with BS EN 197-1:2011 [29] and EN 15167-1:006 [30], respectively, in addition to a commercial silica fume with 15,000 m^2^/kg Blaine fineness, were used in this study. Dune and crushed sand with fineness modulus of 0.48 and 3.2, respectively, were used with a maximum aggregate size of 4.75 mm. Note that these types of sand were selected in the present investigation as these are the commonly used types of fine aggregates in the UAE. The particles size distribution of the aggregates is illustrated in Figure 2. Table 2 summarizes some of the properties of the materials used in the current investigation.

A commercially available superplasticizer (SP) (MasterGlenium SKY 504), complying with ASTM C-494 Type F&G, in addition to a viscosity modifier admixture (VMA) (MasterMatrix 110), were used for the printing concrete production. All mixes were prepared with a constant water-to-binder (w/b) ratio of 0.26. 

### 2.2. Mixing and Printing Procedure 

In phase 1 evaluation, the SP and VMA dosage had to be optimized to obtain the best extrudability results. All mixes were prepared using a Hobart mixer and followed the same procedure. First, all dry ingredients were added and mixed at a low speed of 140 ± 10 rpm for 1 min. Water and superplasticizer were then added and mixed at low and medium speeds (285 ± 10 rpm) for 2–3 min. After approximately 4 min, the VMA dosage was added, and mixing continued for 2 min. The Hobart mixer has proven to be very efficient for mixing concrete with a 0.26 w/b ratio. 

As for the conventional mixer, after the fine aggregates were added to the mixer, additional water was first added to the fine aggregates to account for the water absorption. The mixer was then rotated for 1–3 min, followed by the addition of the rest of the materials. The mixing water was added roughly in around two halves—the first half did not contain any SP, whereas the second half contained all the SP dosage. After achieving a homogenous distribution of all constituents, the VMA dosage was added. The conventional mixer had a speed of 17.5 rpm, and the mixing time to achieve a homogeneous mix was approximately 40 min. It is worth mentioning that the conventional mixer had to be tilted several times to make sure that the water, SP, and VMA had reached all the particles of the mix. This type of mixer was not very efficient for mixing concrete with a 0.26 w/b ratio, and thus it is expected that if the w/b ratio was lower than 0.26, other high-energy mixers would be required. This finding is in line with other studies [3] that show that concrete developed with very low water-to-binder ratios, such as ultra-high performance concrete requiring high energy mixers to be able to produce a fresh workable paste. Additionally, the absence of coarse aggregates in the mix also limits shear stress applied during mixing, and this is why mixes with only fines and low w/b ratio require high-energy mixing. 

**Figure 3:**
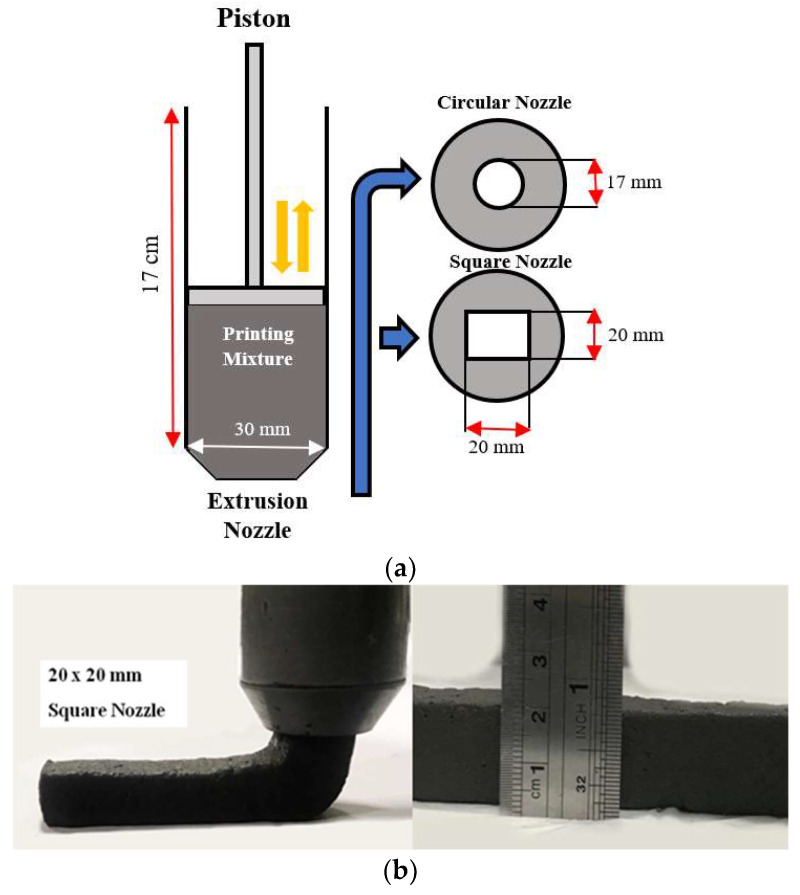
Custom-made nozzle. (**a**) Schematic diagram of manual extrusion; (**b**) square nozzle illustration.

A custom-made 3D printer nozzle was manufactured to simulate an extrusion-based 3D printing process. A piston-type extruder was used, and moderate external vibration was applied to ensure that the mixture is adequately compacted inside the extruder. Once the mixture is filled in the 17 cm circular tube manually, the piston is used, and manual extrusion is performed to print the mortar layers. The pressure was kept constant throughout the extrusion process, and the printing rate was approximately 4.2 cm/s. Two nozzles were used, a circle nozzle with a diameter of 17 mm and a square nozzle with dimensions of 20 × 20 mm. Figure 3a demonstrates a schematic diagram of the nozzle, while Figure 3b demonstrates the extrusion process of the square nozzle.

### 2.3. Tests Conducted

This section explains all the conducted tests along with the devices used. Note that all fresh properties were tested once since it is time-sensitive. However, each mix was repeated at least three times to ensure repeatability of the results.

#### 2.3.1. Extrudability

The first criteria to be investigated for printing concrete is extrudability, which lies in a smooth grading of materials. Le et al. [5] and Ma et al. [17] considered a target length for the extrudability; however, both Malaeb et al. [12] and Kazemian et al. [31] did not consider any target length in the evaluation and focused on the filament shape itself. In the current investigation, for a mix to pass the extrudability criteria, the filament has to maintain the shape of the nozzle and has to be extruded continuously without any separation or blockage. 

#### 2.3.2. Setting Time and Open Time

The setting time was performed for the optimum mix with a/b ratios of 1.2, 1.5, and 1.8 as per ASTM C191-19 [32]. The setting time was determined for all mixes prepared using both the Hobart mixer and conventional mixer to ensure that both mixing methods led to the same setting time results. The open time, on the other hand, is the time at which the filaments are no longer extrudable. To determine the open time of the mixes, a filament was extruded every 15 min until blockage or separation occurred. 

#### 2.3.3. Workability

**Figure 4:**
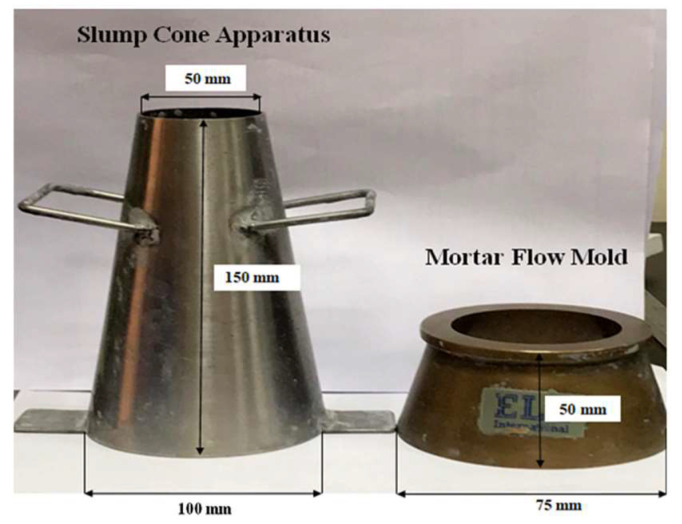
Slump cone and mortar mold dimensions.

Two methods were adopted to study the workability of the printing concrete. The first test method involved performing the slump test. The standard slump cone has an internal diameter of 200 mm at the base and 100 mm at the top and a height of 300 mm. These dimensions are suitable for concrete that has an aggregate size of up to 37.5 mm. Since the concrete used in this study has a maximum aggregate size of 4.75 mm and could be considered as mortar, a cone with half the dimensions of the original slump cone was proposed. The base and top diameter and the height of the proposed cone are 100, 50, and 150 mm, respectively, as shown in Figure 4. These dimensions were also used in [14]. The slump test was performed every 15 min until the concrete was no longer extrudable. The test indicates the homogeneity, consistency, and buildability of the concrete mix. 

The second test method selected was the flow of mortar test that was performed as per ASTM C1437-20 [33]. The flowability was estimated based on the spread of mortar subjected to 25 drops in 15s of the flow table using the caliper as per ASTM C 230-21 [34], the diameter is measured along the four lines scribed at the tabletop, and the flow is recorded in percentage (%) as the total of the four readings. The flow test provides a better indication of the rheological properties of the concrete as compared to the slump test since it measures the resistance of the concrete to flow within a specific time, 15s in this case. As in the slump test, the flow test was performed every 15 min until the concrete was no longer extrudable. 

#### 2.3.4. Buildability

The ability of the printed layers to maintain their shape once extruded and bear the pressure exerted by the upper layers demonstrates the buildability. It is the vertical stacking of layers. Several studies have followed different approaches to assess shape stability (buildability). Some evaluated it by either determining the maximum number of layers that can be printed without noticeable deformation [5,12] or by measuring the vertical settlement of the printed layers [13,35]. Others also attempted to propose models to predict the collapse of the printed structure [17,36,37]. In the current study, this criterion was assessed by determining the maximum number of layers that can be printed without noticeable deformation using circular and square nozzles. 

#### 2.3.5. Compressive Strength

**Figure 5:**
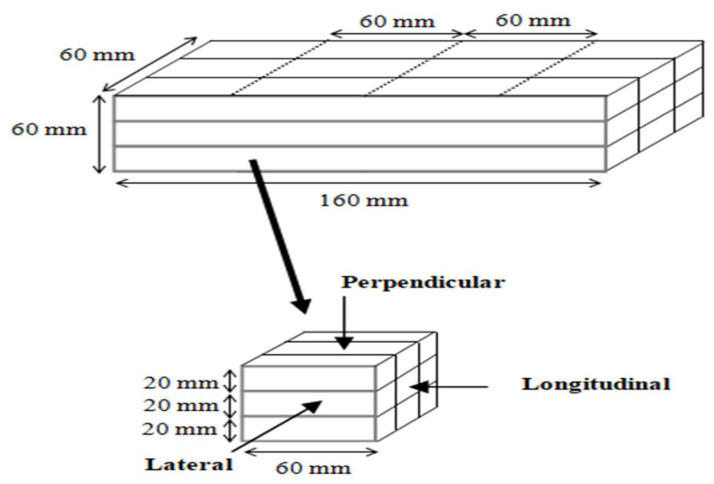
Cutting diagram and testing direction of printed concrete.

The compressive strength was performed on all mixes of phase 1 and 2 evaluations. In phase 1, the strength of cast specimens of the two groups was tested at 3 days to have an indication of the concrete compressive strength while choosing the optimum mix. In phase 2, the compressive strength of both cast and printed specimens was performed mainly to indicate the effect of the layering process on the concrete. The compressive strength was performed as per ASTM C109-20 [38]. The cast specimens had a cube size of 50 × 50 × 50 mm, and three specimens were tested for each mix. The dimensions of the printed specimens were 60 × 60 × 60 mm, which were prepared from 160 × 60 × 60 mm printed samples, as shown in Figure 5. At least two specimens were tested in the perpendicular direction for each mix.

## 3. Results and Discussion

The results and discussions are organized based on the different phases of the investigation. The results in phase 1 are presented and discussed with respect to the extrudability, compressive strength, and setting time of the mixes using the Hobart mixer. On the other hand, the results in phase 2 are discussed based on the extrudability, setting time, open time, workability, buildability, and compressive strength of the mixes with different aggregate to binder (a/b) ratios (1.2, 1.5, and 1.8) using the conventional mixer. 

### 3.1. Phase 1 Evaluation

#### 3.1.1. Group I

**Figure 6:**
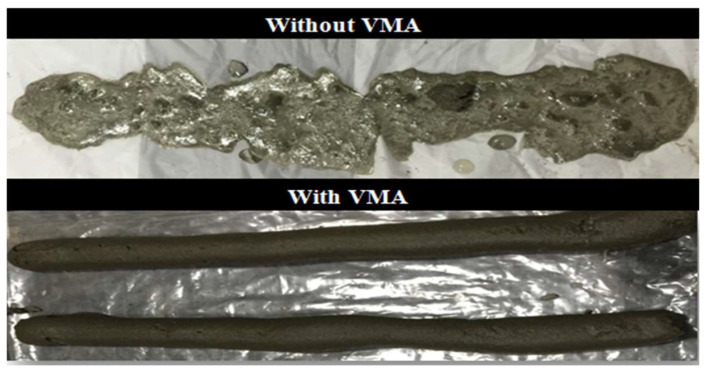
Control mix extrudability.

The extrudability of the D-CM and D-GB mixes was the most difficult to adjust. Several trials were performed to achieve acceptable extrudability of the control mix (D-CM). VMA was initially not used in the control mixes to prevent additional costs. This mix failed as it was too stiff to extrude through the nozzle. Additional superplasticizer was added to adjust this mix; however, segregation occurred as a result and the mix became too flowable which led to blockage. Most of the mixes segregated or became unstable when either the water-to-binder ratio or the SP were increased; however, when VMA was used, segregation did not occur and extrudability was achieved, as shown in Figure 6. The VMA dose used in this study was 0.24% by total weight of fines. Similarly, the D-GB mix was also difficult to extrude, and the use of VMA was essential to adjust its extrudability. This mix was to an extent easier to extrude than the D-CM mix. The increased cohesiveness achieved by the addition of GB improved the extrudability performance. Moreover, the SP dosage used to adjust the extrudability of the D-GB mix was lower than that of the D-CM mix. This was expected as, unlike cement, only a few water molecules get absorbed by the GB particles due to their dense surface, thus enhancing its workability [3,39]. 

The use of a low w/b ratio in either the control mix or the D-GB mix caused the mix to be too stiff and pumpability and extrudability could not be achieved, and hence it was necessary to increase its flowability. The increase in flowability for extrusion can either be achieved by increasing the w/b ratio or SP dosage. This, in turn, may either cause the mix to segregate or result in unstable filaments. Therefore, the use of VMA is essential with the increase in w/b ratio or SP to adjust the viscosity of the mix and to account for the excessive water, which may increase the chances of blockage. As expected, the D-SF mix was much easier to extrude than the D-CM and D-GB mixes. The use of SF is well known to increase the cohesiveness of the mix which consequently improves the pumpability of the concrete and enhances its extrudability. To obtain the combined benefit of each of the GB and SF, a mix containing both materials was developed (D-GBSF).

#### 3.1.2. Group II

**Figure 7:**
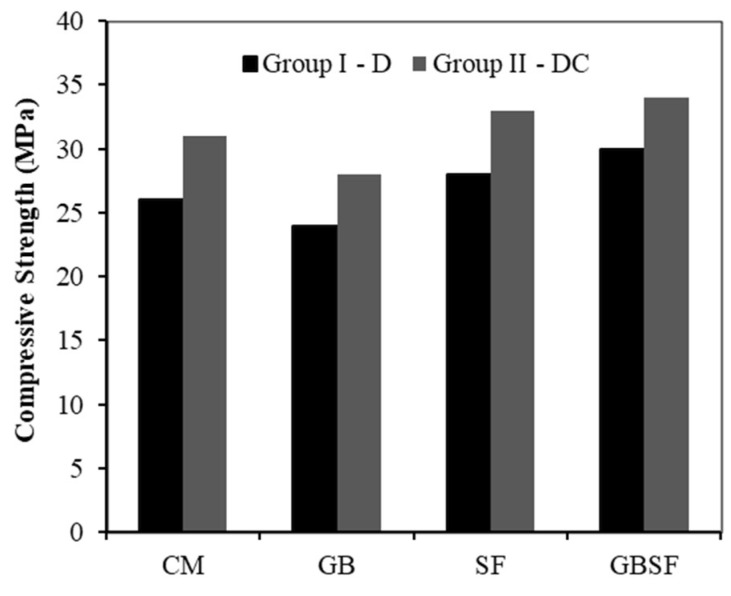
Cube compressive strength at 3 days for Group I and II mixes.

Similar to Group I, the use of GB and SF had the same effect on Group II mixes. The DC-CM and DC-GB mixes were the most difficult mixes to adjust for extrusion as compared to the DC-SF and DC-GBSF. The use of crushed sand along with dune sand facilitated the extrusion process for the four mixes as compared to the mixes of Group I. This is mainly attributed to the production of a smoother gradation curve, which thereby enhanced the extrusion performance. Figure 7 shows the cube compressive strength at 3 days for Group I and II mixes. The DC-GBSF mix had the highest strength of 34 MPa at 3 days, which was quite similar to the D-SF mix. The DC-GBSF mix originally had a 1.2 a/b ratio and was further developed to have ratios of 1.5 and 1.8. Table 3 shows the initial setting time (IST) of the 1.2, 1.5, and 1.8 mixes using the Hobart mixer. As observed, the initial setting time decreases with the increase in a/b ratio. Cold joints may develop between the layers if the time gap exceeds the IST. However, the formation of cold joints can even occur due to other reasons before the IST, as will be discussed later. 

### 3.2. Phase 2 Evaluation 

In this phase, all fresh properties in addition to the compressive strength of the mixes produced using the conventional mixer are discussed. 

#### 3.2.1. Setting Time and Open Time 

**Figure 8:**
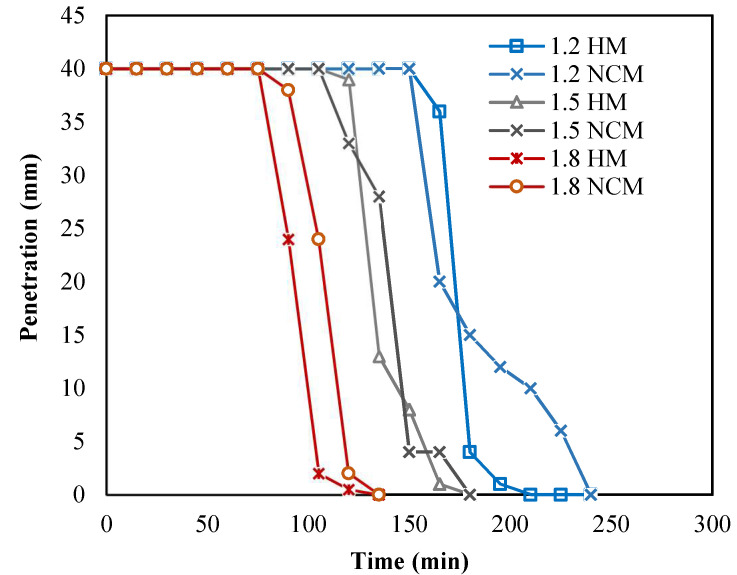
Penetration vs. time results of Hobart mixer (HM) and conventional concrete mixer (NCM).

As previously mentioned, the mixing speed for the conventional mixer was 17.5 rpm, which is considered relatively slow compared to the Hobart mixer. The mixing speed is one of the factors that affect mixing time, and thus several trials were first conducted to achieve the optimal mixing time while utilizing the conventional mixer. During the first trial, the mixing time was approximately around 20 min, and the mix was too stiff to extrude. Therefore, the mix required an increase in workability that could easily be achieved by increasing the SP dosage and/or the w/b ratio. However, to avoid any changes in the mix proportions, other factors were optimized such as the mixing time to account for the change in mixing speed/technique. The results show that when the mixing time was increased to 40 min, the mix was extrudable and the setting-time results were analogous to that of the Hobart mixer (≤15 min. difference), as illustrated in Figure 8, and thus the SP dosage and w/b ratio of the mixes were left unchanged. Table 3 presents the initial setting time from both phases and open time results of the conventional mixer from phase 2. As shown from the table, the open time was lower than the IST for the three mixes, which emphasized the importance of the open time for printing concrete. Moreover, the results also showed that the IST and open time decreased with the increase in a/b ratio. 

#### 3.2.2. Comparison of Workability Indicators with Extrudability and Structuration Rate

**Figure 9:**
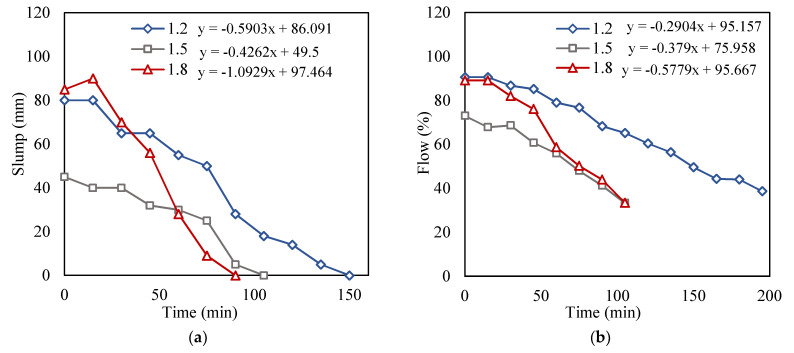

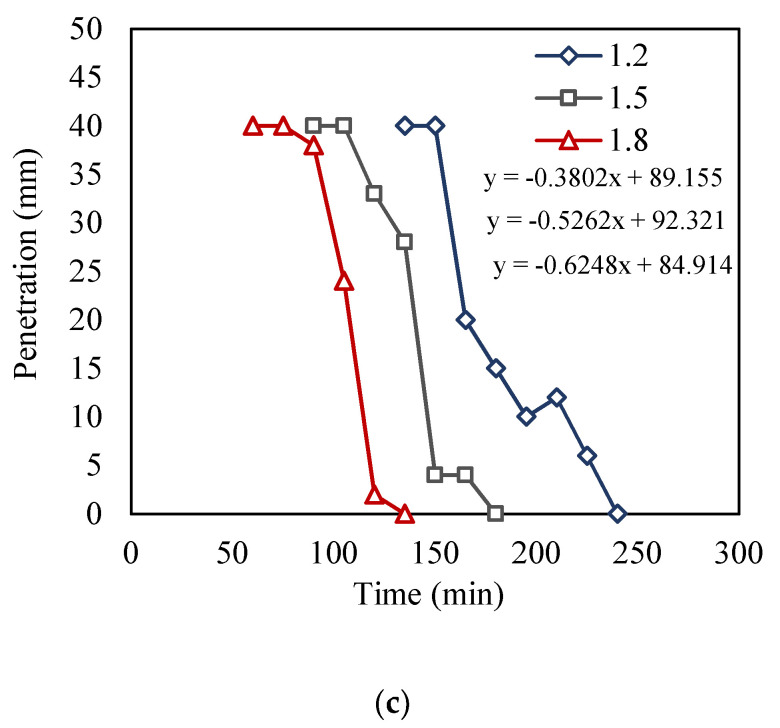
Change in workability with time. (**a**) Slump; (**b**) flow; (**c**) penetration.

The workability results of the conventional mixer of the 1.2, 1.5, and 1.8 mixes are shown in Figure 9. The penetration was also monitored with the workability as it indicates the loss of workability with time. Each of the slump, flow, and penetration was done simultaneously along with the extrudability to determine the loss of workability with time. The equations included in Figure 9 demonstrate the slopes of the curves, which will later be discussed in this subsection. 

Slump test: From Figure 9a, it can be shown that at 0 min, the three mixes had a slump ranging from 85 to 50 mm. The times at which the 1.2, 1.5, and 1.8 mixes were no longer extrudable were determined previously as 135, 90, and 75 min, respectively, as listed in Table 3. These values corresponded to a slump of 5, 5, and 9 mm, respectively, indicating that the slump range to satisfy the printing quality fell in the ranges of 85 to 9 mm. It was reported in [14] that the slump range that maintained printability lay within a range of 88 to 32 mm. The upper range in [14] complies with the results of the current study. Nevertheless, for the lower range, slump values lower than 32 mm were also found to provide acceptable extrudability. This was perhaps because the lower range of the slump values is more sensitive to the power of the 3D printer or pressure exerted, which differ on the basis of the properties and type of printer used. This can be a possible premise as to why the lower range of workability varies between different studies as opposed to the upper range. Additionally, the 1.8 slump values were found to be intermediary between the 1.2 and 1.5 values; this was possibly due to the use of VMA, which was used by weight of total fines rather than the total weight of cementitious materials. 

**Figure 10:**
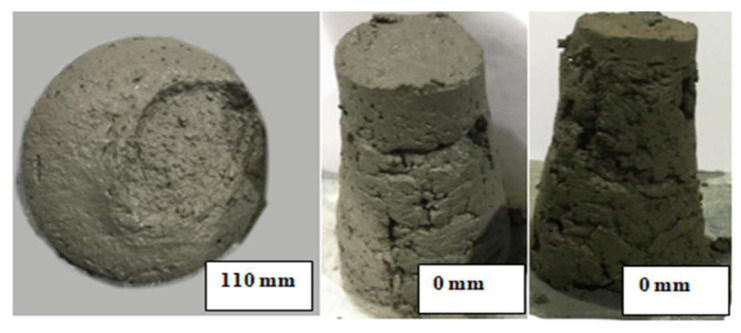
Unacceptable slump.

Concrete with higher slump values (>90 mm) led to unstable filaments. As for the 0 slump, there were times at which the 0 slump provided acceptable extrudability and times at which the filaments were discontinuous and difficult to extrude. Hence, it can be concluded from the slump results that the slump only provides an indication of the extrudability, consistency, and homogeneity of the concrete, and cannot be completely relied on to evaluate the workability of the printing concrete. Extrudability has to be within a slump ranging from 85 to 9 mm; however, it is worth noting that these values will not always necessarily cause the concrete to pass the extrudability criteria, and hence other test methods should be proposed to evaluate the workability of the printing concrete. Figure 10 demonstrates concrete with a 110 mm slump, which failed the extrudability criteria, as well as 0 slump after the open time of the mixes were exceeded. It is clear that at this stage, the concrete lost some of its plasticity and will no longer produce concrete with the acceptable printing quality. Even if the concrete was extruded, cold joints may occur, and bonding between the layers may be very weak. Therefore, the lower range of the workability should also account for the adequate bond strength between the printed layers. 

**Figure 11:**
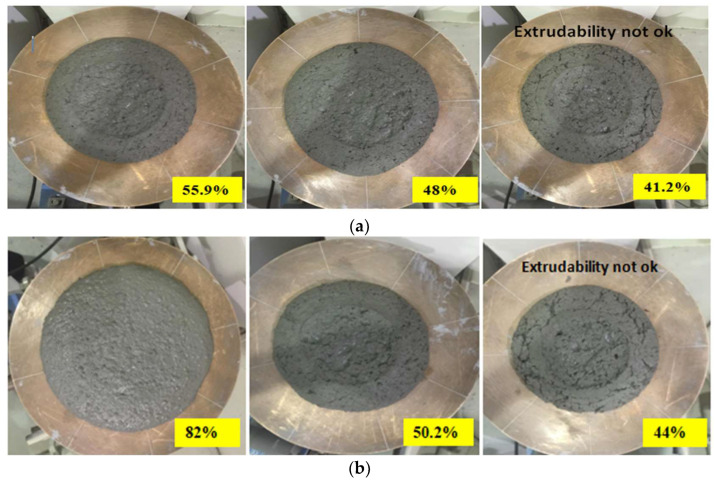
Mortar flow test lab results. (**a**) Flow of 1.5 mix; (**b**) flow of 1.8 mix.

Mortar flow test: From the mortar flow test results, when the flow was around 56.4% and lower, the extrudability was affected. Results also showed that when the flow was beyond 95%, the filaments were unstable and inconsistent. In Figure 11, some of the flow results of the 1.5 and 1.8 mixes are depicted. As observed, at 41.2% and 44%, the concrete appeared to be stiff with microcracks appearing in the mixture paste. Such performance led to unacceptable extrudability with separation and discontinuity in the printed layers. In general, the flow test provided more consistent results as compared to the slump test and showed a better indication of the structuration rate. The 25 drops of the flow test in particular were very useful to reveal the loss of viscosity with time. The microcracks appeared as a result of the loss of viscosity, indicating the concrete is no longer homogenous and will lead to the formation of a weak layer interface. Similar to the slump results, from Figure 9b, it can be shown that the flow results of the 1.8 were also intermediary, and this could be attributed to the use of VMA by total weight of fines as previously mentioned. 

Structuration rate: The slopes of the curves shown in Figure 9 demonstrate the rate at which workability was lost with time. The rate at which the mixes stiffened indicates the structuration rate. The slopes of the 1.2, 1.5, and 1.8 mixes for the slump (Figure 9a) were found to be −0.59, −0.42, and −1.09, respectively, which can be read from the equations of Figure 9a. The steeper the slope, the higher the structuration rate, and vice versa. From the results, the 1.5 mix showed a lower slope (−0.42) than that of the 1.2 mix (−0.59), indicating that the structuration rate for the 1.2 was higher than that of the 1.5 mix. This was unexpected, as the aggregate to binder ratio is a factor that affects the structuration rate of the mixes, and it is expected that, as the a/b ratio increases, the structuration rate should also increase. Thus, this suggests that the slump test may not always provide accurate results. 

Figure 9b, on the other hand, showed that as the a/b ratio increased, the slopes became steeper, i.e., the structuration rate increased with the increase in a/b ratio. Similar to the flow time results, the penetration time results also showed that as the aggregate-to-binder ratio increased, the structuration rate increased, as depicted in Figure 9c.

#### 3.2.3. Pearson Correlation Analysis 

This method was adopted to find the relationship strength between each of the workability indicators with time as well as their mutual relationships to confirm the findings of the study. Since extrudability is a time-dependent factor, it is critical for the workability indicators to have a very strong relationship with time. Table 4 demonstrates the Pearson correlation coefficient of each of the 1.2, 1.5, and 1.8 mixes. 

When comparing each of the variables with time, all the coefficients had a negative value, indicating that as time increased, there was a loss in workability. Flow had the strongest relationship with time as the mixes had coefficients ranging from 0.98 to 0.99, followed by the penetration and slump, which had coefficients ranging from 0.87 to 0.91 and 0.76 to 0.89, respectively. Slump had the lowest relationship strength with time as compared to penetration and flow.

From the slopes of the penetration and flow (Figure 9b,c), the structuration rate was found to increase as the a/b ratio increased. However, the slump showed that the 1.5 mix had a lower structuration rate than that of the 1.2 mix. From the correlation analysis performed, the slump had the lowest correlation with time when compared to flow and penetration. This indicates that the structuration rates obtained from the slump results may have been less accurate as compared to the rates obtained from the penetration and flow. On the basis of the statistical analysis, it is observed that the flow test would provide the best indication of structuration, followed by the penetration and slump tests.

#### 3.2.4. Buildability 

This property was evaluated for the three mixes using both the circular and square nozzle. Buildability is dependent on all the factors that affect the structuration rate as well as the shape of the layers, which plays a role in providing stability to the structure. The factors that affect the structuration rate are the mix proportions, a/b ratio, w/b ratio, and materials used. Each of these has a significant impact on buildability. For example, it was shown in [37] that an increase in superplasticizer (SP) dosage or water to cement content reduces the thixotropy of concrete, which in turn reduces the buildability performance. 

From the results of this study, it was observed that as the a/b ratio increased, the buildability also increased, regardless of the shape of the nozzle. It is logical that if the mixes had similar flow (green strength) initially, the difference in buildability would not be detected during the first couple of minutes. However, as time increases beyond a few 10s of minutes, the rate of structuration (thixotropy) will play a role, and the layers of the mixes with lower a/b ratio will lose their shape stability upon extrusion as they have lower structuration rates and therefore would not have enough time to gain sufficient strength. For this study, within the first 15 min, all mixes had the same number of layers (10 layers) using the square nozzle. After approximately 15 min, the buildability for each mix varied. 

**Figure 12:**
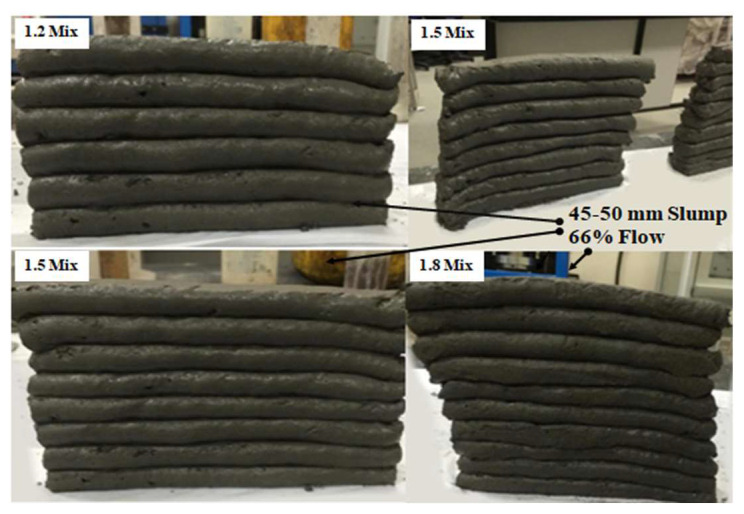
Buildability of the 1.2, 1.5, and 1.8 mixes using the circular nozzle.

As previously stated, from the penetration and flow results, the structuration rate was highest for the 1.8 mix, followed by the 1.5 and 1.2 mixes. Thus, it was expected for the 1.8 mix to have the highest number of layers within the shortest time since it had the highest structuration rate. It is important to mention that the filaments were extruded from the same batch continuously with a time gap of approximately 1.5 min. The 1.8 mix showed the best performance, followed by the 1.5 and 1.2 mixes. The maximum number of layers reached by the circular nozzle was 10 layers for the 1.8 mix. External supports were required to go beyond 10 layers, which is because circular nozzles, in general, do not provide good shape stability and have a low area of contact compared to square and rectangular nozzles. The buildability of the circular nozzle of each of the 1.2, 1.5, and 1.8 mixes is demonstrated in Figure 12.

**Figure 13:**
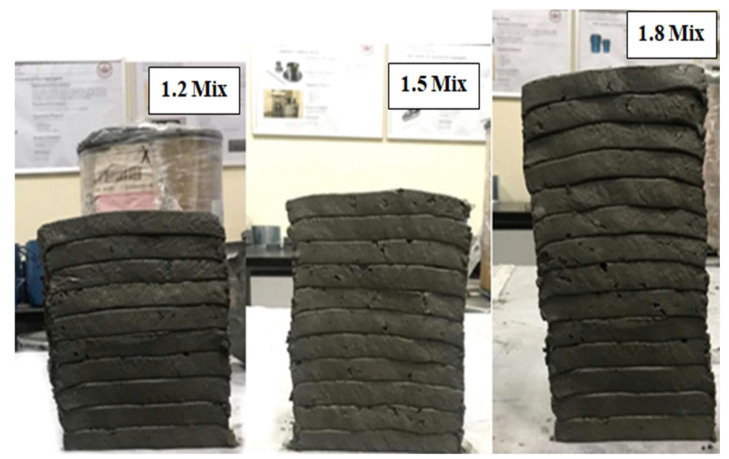
Buildability of the 1.2, 1.5, and 1.8 mixes using the square nozzle.

**Figure 14:**
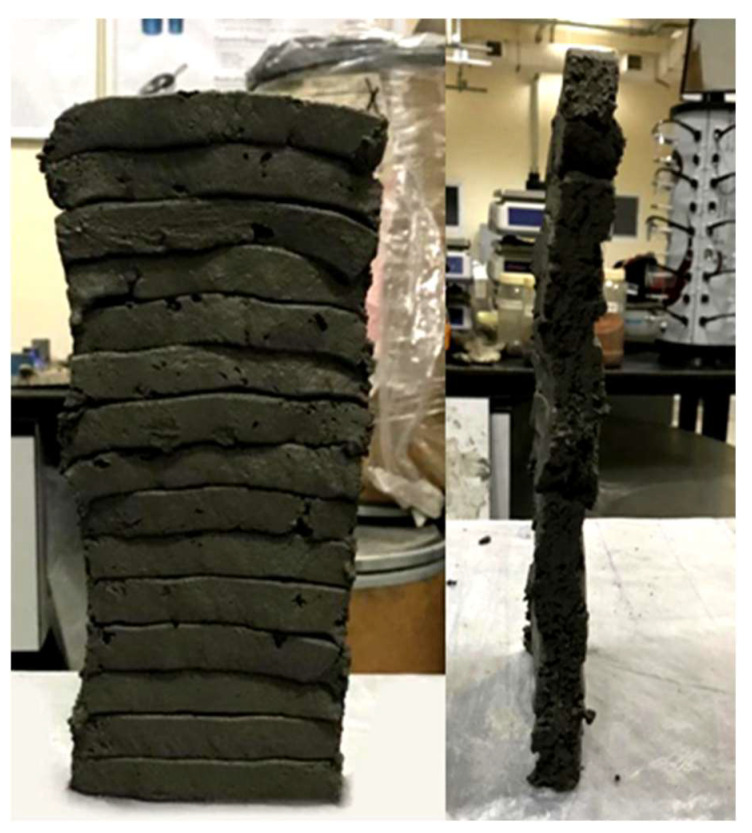
Buildability of the 1.8 mix with 15 layers.

The 20 × 20 mm square nozzle produced more stable filaments compared to the 17 mm circular nozzle. The buildability of the square nozzle for the three mixes is shown in Figure 13 and Figure 14. For the 1.2 mix, up to 10 layers were reached; after that, due to the pressure of the upper layers on the lower layers, the shape became unstable, and external supports were required to proceed further. The 1.5 mix reached up to 11 layers, and again external supports were required (see Figure 13). As for the 1.8 mix, up to 15 layers were reached without external supports, and no failure occurred, as shown in Figure 14. 

It is worth mentioning that the workability of the mixes had to be adjusted to satisfy the buildability requirement. The computed slump and flow range to maintain printability were found to be within the ranges of 85 to 9 mm and 90.6 to 56%, respectively. These values provide good extrudability performance but do not satisfy the buildability requirement. The buildability demonstrated in Figure 12, Figure 13 and Figure 14 corresponded to slump and flow ranges of 45–55 mm and 66–60%, respectively.

#### 3.2.5. Compressive Strength 

**Figure 15:**
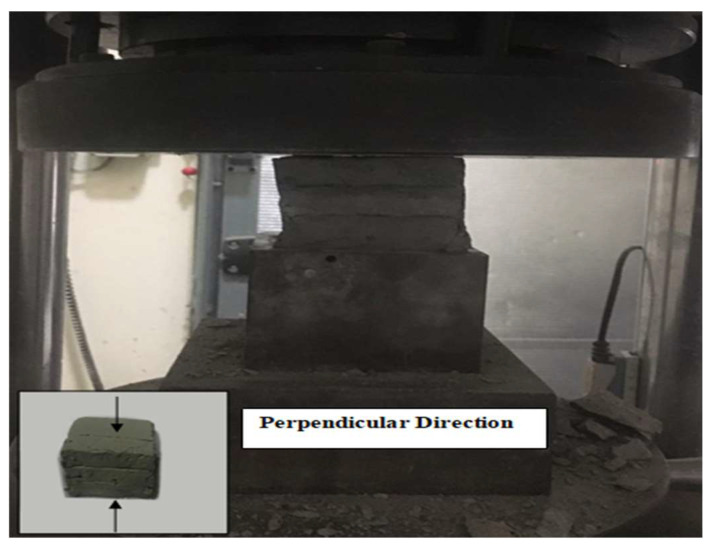
Compression strength test set-up.

**Figure 16:**
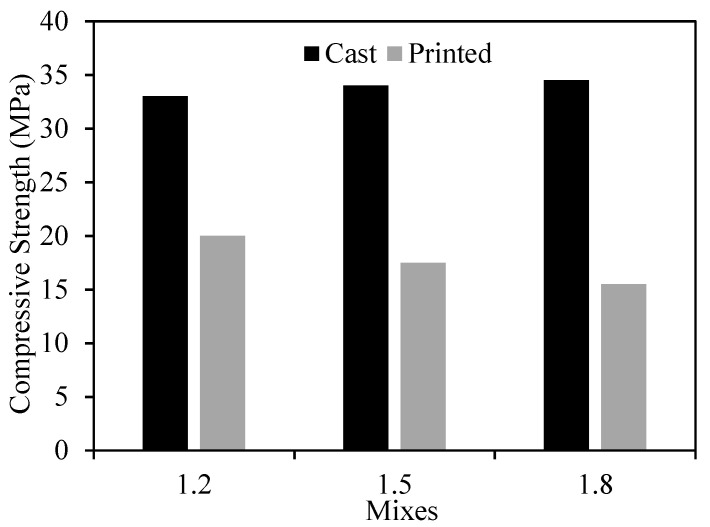
Compression strength of cast and printed specimens at 3 days.

**Figure 17:**
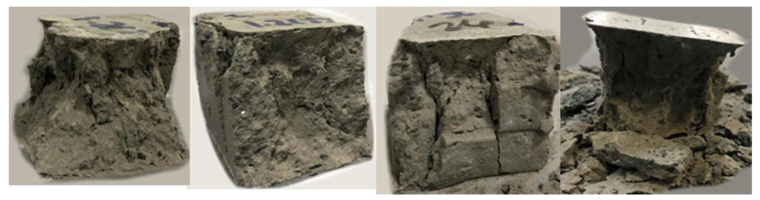
Failure modes of 3 × 3 layer printed specimens.

The strength of the cast specimens of the 1.2, 1.5, and 1.8 mixes were 33, 34, and 34.5 MPa at 3 days, and 44.3, 52, and 53.8 MPa at 28 days, respectively. The results show that the compressive strength increased with the increase in a/b ratio. In Figure 15, the testing of the 3 × 3 layer cube is illustrated. The compressive strength of the cast and printed specimens at 3 days is presented in Figure 16. Figure 17, on the other hand, shows the failure modes of the printed specimens. As observed, the layering process in this study did not affect the failure modes of the concrete cube specimens, and most exhibited a semi-explosive failure mode. The specimens witnessed acceptable failures that are similar to those of the cast specimens. Moreover, debonding was not detected between the layers of the printed cubes upon compression.

For the printed specimens, the compressive strength decreased with the increase in a/b ratio. These results are contrary to the cast specimen results. Although high thixotropy is usually preferred for printing concrete for buildability purposes, as previously shown, several studies have reported that increased thixotropy can also lead to weak bonding between the layers and cause cold joints [16,40]. In fact, in [16], it was noted that a weaker interface between the layers can occur as a result of longer waiting time and/or increased thixotropy. Therefore, since the printing time for the cubes of all mixes was not changed, and according to literature results, the decrease in strength witnessed in this study for the printed cubes could be attributed to the increased thixotropy, which occurred as a result of an increase in a/b ratio. 

The results also show that the printed specimens exhibited lower strength than that of the cast specimens, which is in line with previous studies [41,42,43,44]. One of the possible justifications to the strength reduction could presumably be due to the time required to print the cubes. Although the cube printing occurred during the open time, the bonding between the layers may have weakened. This denotes that the open time of printing concrete should be selected as the time, which also maintains sufficient interlayer strength. Another probable reason for the strength reduction could have been due to the imperfect shape of the cube. While extracting the 60 mm cubes from the 160 × 60 × 60 mm printed samples, the cube edges were slightly sloping. This might have contributed to the reduced strength of the printed specimens.

#### 3.2.6. Optimum 3D Printing Concrete Mix 

Table 5 provides a summary of phase 2 results using the conventional mixer. Overall, the results of the study showed that the aggregate-to-binder ratio (a/b) had a direct impact on the structuration rate of concrete, which affects the buildability and compressive strength of printing concrete. The increase in a/b ratio affects the availability of cement for binding, which in turn affects the structuration rate of concrete. Although the buildability was enhanced with the increase in a/b ratio, the compressive strength of printing concrete was adversely affected due to an increase in structuration, which might have weakened the interlayer strength. This indicates that higher structuration rates are not preferred for printing concrete. As the a/b ratio is one of the factors that affect the structuration rate, high a/b ratios might not be suitable for 3D printing. Therefore, from the compressive strength results, it is suggested that, overall, the 1.2 mix is the optimal mix to avoid interlayer strength loss, as well as to limit the extent of variation between cast and printed specimens. It is worth mentioning that in this study, the a/b ratio was not decreased beyond 1.2, as this will increase the cement and SF contents that are not desired from environmental and economical perspectives. Increasing the cement and SF content can also increase the heat of hydration, which consequently will increase the susceptibility of concrete to shrinkage. 

Moreover, enhancing the buildability of the 1.2 mix is more controllable as opposed to enhancing the interlayer strength of the 1.5 and 1.8 mixes. It can visually be seen from Figure 14 and Figure 15 that there was no significant deformation in the very first layer of the mixes. This denotes that the size of the nozzle played a major role in the buildability failure of the mixes. If a wider rectangular nozzle was used, the buildability of all mixes would have certainly improved as a result of an overall enhancement in the shape stability of the printing element. Other methods to enhance the buildability without altering the structuration rate include the addition of external supports or increasing the paste age. However, increasing the paste age is not always a feasible solution as it could lead to superficial drying, which in turn reduces the interlayer strength [44]. Therefore, from this perspective, it is suggested that the optimal mix is the 1.2 mix, which will be selected for future investigations.

## 4. Conclusions

The main goal of the present investigation was to develop 3D-printing concrete using local construction materials and study its workability and structuration rate through employing commonly used devices. The critical early age properties studied included the extrudability, setting time, open time, workability, and buildability of printing concrete in addition to the compressive strength. The following conclusions are drawn from the study:

- Mixing using the conventional mixer requires more time as compared to the Hobart mixer. The mixing time was significantly increased from 5 min on the Hobart mixer to 40 min on the conventional mixer as a result of changes in mixing speed/technique. This emphasizes the importance of optimizing the mixing time when using different concrete mixers. Overall, the conventional mixer was adequate to prepare mixes with 0.26 water-to-binder (w/b) ratio; however, for lower ratios, other high-energy mixers may be required.
- The workability results suggest that the optimal ranges to satisfy the extrudability are in the ranges of 85–9 mm and 90–56%, respectively. However, the lower limit may differ from one study to another, depending on the properties of the printer used.
- The structuration rate was indicated by the loss of each of the flow, slump, and penetration with time. From the Pearson correlation analysis results, it is suggested that flow and penetration provide the best indication of the structuration rate of concrete as compared to slump, and thus it is recommended to correlate these results with the rheological properties.
- The higher aggregate-to-binder (a/b) ratio increased both the buildability and compressive strength of cast specimens. However, for the printed specimens, the strength decreased with the increase in a/b ratio. The decrease was mainly due to an increase in thixotropy. This suggests that the optimal mix in this study was the 1.2 mix, and higher a/b ratios are not preferred for 3D printing or have to be used alongside other materials that lower the thixotropy to avoid the formation of weak interfaces.

From this study, it is concluded that flow and penetration show a good indication of the workability of printing concrete, and thus future research will be devoted to studying the correlation between each of these tests with the rheological parameters (yield stress, viscosity, and thixotropy). The results of the present investigation show that simple devices can be used to describe some of the rheological properties of concrete, and therefore this can encourage researchers to evaluate the rheological properties using simpler devices that are currently needed due to the complexities associated with concrete rheometers. Additionally, from the lab observations, it is clear that the interlayer strength is also affected by different workability results, and the open time should not only be selected as the time that maintains acceptable extrudability, but also as the time that maintains adequate interlayer strength. Therefore, future research will be focused to study the effect of different factors on the interlayer strength of printing concrete.

## Figures and Tables

**Table 1:** Summary of optimum ranges of extrudability for 3D printing mixes.

Reference	Test Method	Description/Standard	Acceptable Range
Le et al. [5]	Shear vane test	A 90 mm diameter vane was used and the shear strength was determined by measuring the torque as per BS 1377-9:1990	0.3 to 0.9 KPa
Malaeb et al. [12]	Slump-flow	Measured the spread diameter over time as per ASTM Standard C1611/C1611M-14	1.0–2.0 cm/s
Zhang et al. [18]	Drop table test	N/A	192.5–294 mm
	Concrete rheometer	Measured the yield stress, viscosity, and thixotropy	Yield stress: 178.5–359.8 Pa Viscosity: 3.8–4.5 Pa.s Thixotropy: 6284.5 Pa/s
Ma et al. [14]	Slump test	GB/T14,902-2012	88–32 mm
	Jumping table test	GB/T 2419-2005	174–210 mm
	Penetration resistance	GB/T 50,080-2002	13–40 kPa
Rahul et al. [23]	Vane shear test	A four-bladed vane with a diameter and height of 12 and 24 mm was used; torque was determined and converted to yield stress by using the equation of Dzuy and Bogers [24]	1.5–2.5 kPa

**Table 2:** Physical properties of fine aggregates.

Materials	Blaine Fineness (m^2^/kg)	Fineness Modulus	Specific Gravity	Water Absorption (%)
Dune sand	NA	0.48	2.58	2.20
Crushed sand	NA	3.20	2.57	1.00
OPC	318	NA	3.14	NA
SF	15,000	NA	2.20	NA
GB	417	NA	2.91	NA

**Table 3:** Initial setting and open time of a/b ratio 1.2, 1.5, and 1.8 mixes.

	1.2	1.5	1.8
IST—Hobart mixer (min)	170	129	90
IST—Conventional mixer (min)	160	135	105
Open time—Conventional mixer (min)	135	90	75

**Table 4:** Pearson correlation coefficients with respect to time.

Mix	Slump	Flow	Penetration
1.2	−0.89	−0.99	−0.91
1.5	−0.83	−0.98	−0.91
1.8	−0.76	−0.98	−0.87

**Table 5:** Summary of the properties of phase 2 mixes.

	Mixes		
	1.2	1.5	1.8
**Fresh properties**			
Setting time (min)	170	129	90
Open time (min)	135	90	75
**Workability to satisfy extrudability criteria**			
Slump (mm)	80–0	45–5	85–0
Flow (%)	90.6–56.4	73–41.2	89.1–50.2
**Workability used for buildability criteria**			
Slump (mm)	45–50		
Flow (%)	66–60		
**Buildability**			
No. of layers using circular nozzle	6	8	10
No. of layers using square nozzle	10	11	15
**Compressive Strength (MPa)**			
Cast specimens at 3 days	33	34	34.5
Cast specimens at 28 days	44.3	52	53.8
Printed specimens at 3 days (tested in perpendicular direction)	20	17.5	15.5

## Data Availability

The data presented in this study are available on request from the corresponding author.
